# Supplementary material for: Host Serine Proteases and Antiviral Innate Immunity as Potential Therapeutic Targets in Influenza A Virus Infection-Induced COPD Exacerbations
Source: Int J Mol Sci. 2025 Mar 12;26(6):2549. doi: 10.3390/ijms26062549 (PMC11941970; doi:10.3390/ijms26062549)
Supplement: Supplementary file 1 [file ijms-26-02549-s001.zip › ijms-3474130-supplementary.pdf]

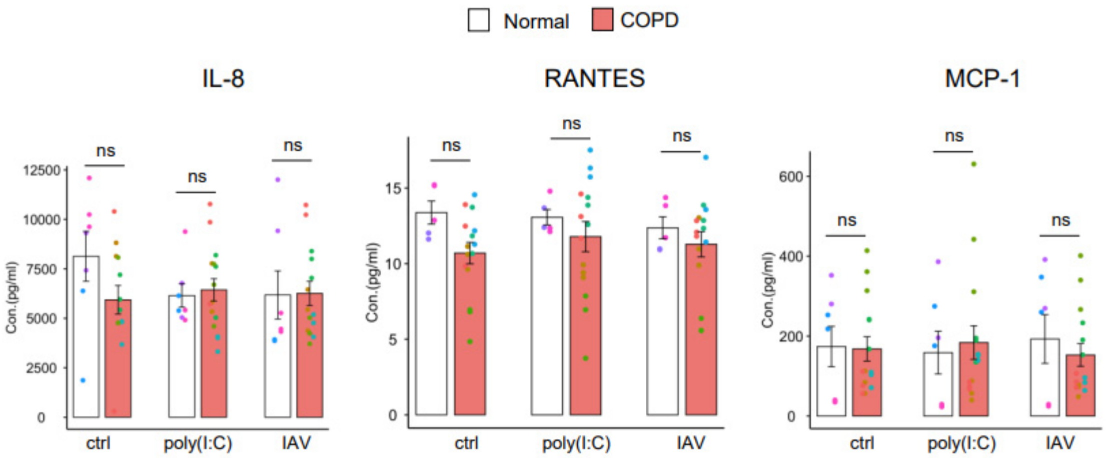

Figure S1. Cytokine production measured in the basal medium. ANOVA and Šidák multiple comparisons test. ns, not significant.

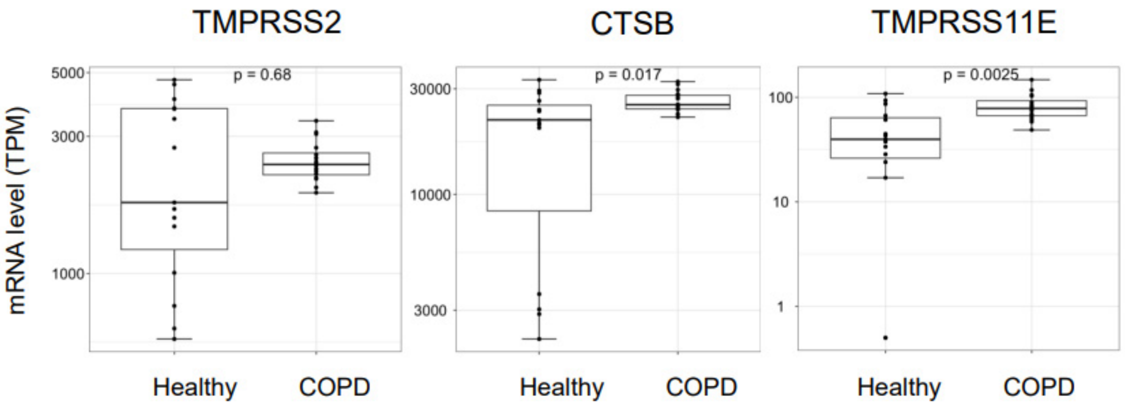

Figure S2. mRNA levels of influenza-activating host protease TMPRSS2, CTSB and TMPRSS11E. Mann-Whitney test.

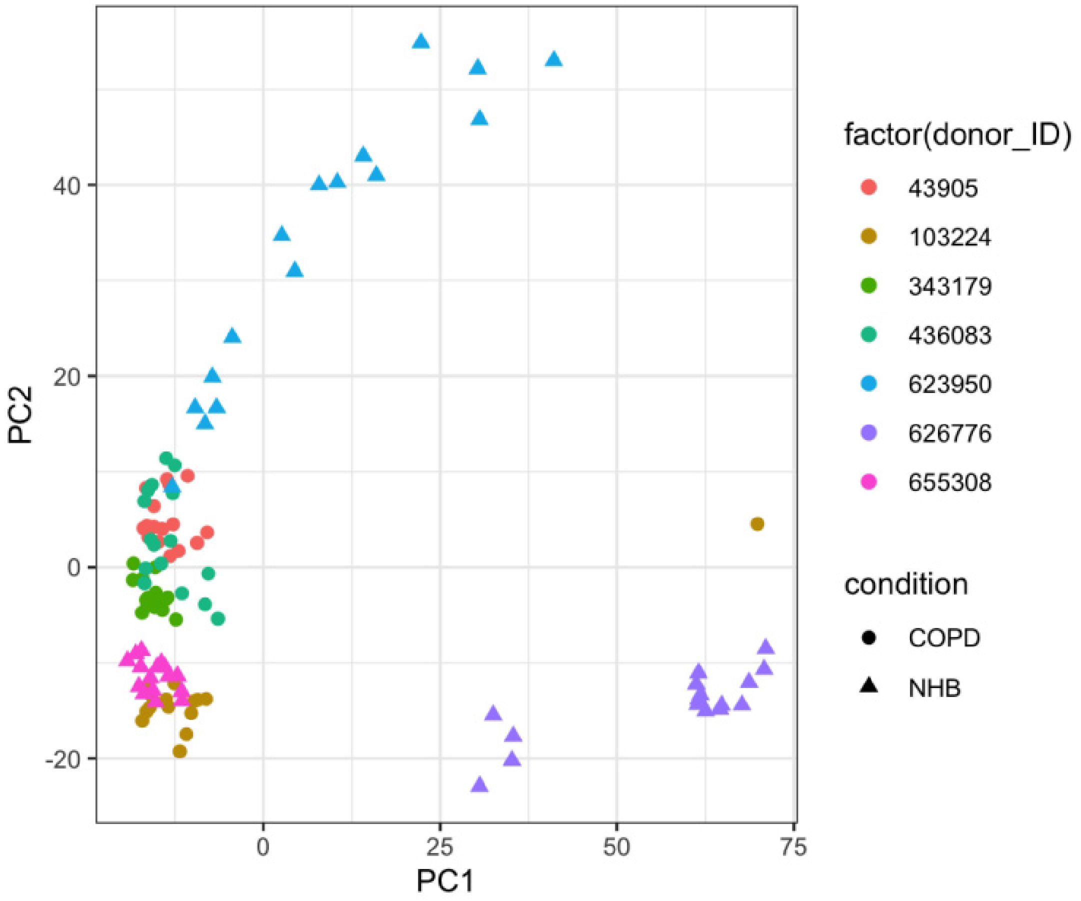

Figure S3. Principal component analysis (PCA) showing sample variations among healthy and COPD donor epithelium.

Table S1. Differentially expressed genes in COPD epithelium.

**Cilia Movement**			**DNA Repair**			**Cell-Cell Adhesion**			**Innate Immune Response**		
**Gene**	**log2FoldChange**	**P-adj**	**Gene**	**log2FoldChange**	**P-adj**	**Gene**	**log2FoldChange**	**P-adj**	**Gene**	**log2FoldChange**	**P-adj**
ZBBX	-2.601329705	9.74E-09	RAD51AP1	-1.7292067	2.86E-08	ROBO2	-4.8405586	1.83E-13	DOCK10	-2.4142962	3.49E-08
SPAG17	-2.103867879	3.84E-07	BRCA1	-1.6565014	5.53E-08	TRO	-3.0014208	9.61E-08	MSH2	-1.2054547	4.95E-07
VPS13A	-1.619650354	2.27E-06	POLQ	-2.6235288	5.86E-08	MYOT	-3.6276975	2.42E-07	SERPINE1	-1.2358673	5.98E-07
MNS1	-1.897844053	3.15E-06	XRCC4	-1.3386623	5.91E-08	CDH2	-2.5732639	4.64E-07	CIITA	-1.5114784	2.92E-06
DNAH7	-1.65794678	3.25E-06	EID3	-2.8568593	1.65E-07	PCDHGB1	-2.0388585	1.51E-06	EXO1	-1.9134748	3.91E-06
KIF27	-1.719021589	9.82E-06	POLA1	-1.9671923	2.10E-07	FAT3	-2.435301	2.00E-06	PLA2G6	-1.7063834	4.71E-06
DNHD1	-1.877545084	9.99E-06	CLSPN	-2.0108018	2.58E-07	HMCN1	-1.9510602	4.93E-06	IL18BP	-2.02355	1.65E-05
SPEF2	-1.764949147	1.43E-05	RFC3	-1.2612029	2.66E-07	PCDHGA6	-2.051032	5.11E-06	FER1L5	-2.138688	2.38E-05
ARMC2	-1.549714046	1.79E-05	ATRX	-2.0126307	2.72E-07	NEXN	-2.4661366	5.50E-06	LCP1	-1.5160636	3.08E-05
DNAAF1	-2.06027754	2.02E-05	RAD51B	-1.7485228	3.13E-07	CDH11	-2.4854489	1.19E-05	SEMA7A	-1.7195961	4.06E-05
TTC12	-1.144524942	2.34E-05	MSH2	-1.2054547	4.95E-07	SDK1	-1.986637	2.05E-05	PRKD1	-1.2467064	7.17E-05
QRICH2	-1.820835152	2.64E-05	BRCA2	-1.8895167	9.14E-07	PTPRM	-1.134461	5.20E-05	SETD2	-1.1093902	0.00014652
DNAH1	-1.99472478	2.71E-05	MCM6	-1.1143178	9.76E-07	DCHS2	-2.3297648	6.03E-05	SUSD4	-1.1998066	0.00017838
DNAH5	-1.252879588	3.03E-05	REV3L	-1.130392	9.78E-07	CRB1	-1.3754696	0.00010784	LEF1	-1.3118665	0.00022713
CCDC39	-1.735209223	4.66E-05	ESCO2	-1.5545006	1.06E-06	CDH8	-1.7233431	0.00011857	GEM	-1.2837723	0.00025826
OFD1	-1.19589813	6.30E-05	PMS1	-1.7396525	1.27E-06	PCDHGA11	-1.4676072	0.00016245	TRIM52	-1.3679018	0.00032493
FSIP2	-2.634685023	6.84E-05	POLI	-1.3764722	1.47E-06	VCAM1	-1.7900708	0.00028561	C5	-1.2677905	0.00071904
CATSPER3	-2.051567442	8.25E-05	FANCD2	-1.0320584	1.61E-06	PCDHGB4	-1.3406951	0.00029711	IKBKB	-1.0856921	0.00105766
SPAG16	-1.174644582	0.00019799	MCM8	-1.681612	2.12E-06	PCDHGB3	-1.9114177	0.00031354	SKAP1	-1.5232264	0.00105847
DNAH11	-1.502903995	0.00019986	FANCL	-1.4192758	2.42E-06	PCDH9	-1.7339448	0.00036152	CLNK	-1.4609664	0.00158878
DNAH17	-1.624787674	0.00036848	BRIP1	-2.247518	3.15E-06	PCDHGA3	-1.6215946	0.00042747	GSDMB	-1.0846449	0.00179362
CATSPER2	-1.392182513	0.0006652	WDR70	-1.4734265	3.46E-06	PCDHGB6	-1.2120087	0.00058641	HLA-DQB1	-2.2658523	0.00182913
DNAI1	-1.330106321	0.00095324	ATM	-1.482807	3.47E-06	AMIGO2	-1.3806884	0.00065837	IL16	-1.2895596	0.00212342
CCDC114	-1.129359946	0.00240726	RIF1	-1.3064819	3.65E-06	CLDN11	-1.4253399	0.00074526	LAT	-1.25503	0.00333986
HYDIN	-1.044874746	0.00338469	ZRANB3	-1.6053757	3.85E-06	PCDHA13	-1.3317304	0.00083911	IL31RA	-1.5104352	0.01060967
CCDC151	-1.130967883	0.00408914	EXO1	-1.9134748	3.91E-06	CDHR3	-1.1350825	0.00120696	LIF	-1.3921078	0.01583063
CCDC40	-1.032435841	0.00562768	MNAT1	-1.1702809	4.39E-06	PCDHGA9	-1.1796526	0.00124614	ENPP3	-1.1427194	0.01765798
SLC26A8	-1.21961369	0.00725406	REV1	-1.3900045	4.68E-06	PCDHGB7	-1.1495128	0.00131788	MEF2C	-1.5638251	0.01920962
TEKT3	-1.313446747	0.00728256	WDHD1	-1.2844466	4.72E-06	ICAM1	-1.5624317	0.00177624	HCK	-1.5944615	0.02530992
SLC22A16	-2.120030768	0.0141972	RAD54B	-1.7377347	4.85E-06	PCDHA12	-1.59299	0.00233762	CXCL3	-1.0340267	0.03817473
			MCM7	-1.0052489	5.89E-06	DSCAML1	-1.2942567	0.00257505			
			PMS2P5	-1.8287584	6.71E-06	CDH6	-1.5450194	0.00337735			
			SMC6	-1.4221584	6.94E-06	PCDHGA8	-1.6841112	0.00780968			
			RAD52	-1.8084247	7.11E-06	PCDHGA5	-1.444059	0.00808534			
			HELB	-2.0896327	8.45E-06	MIR221	-1.2159211	0.01144615			
			DNA2	-1.4263975	8.46E-06	PCDHGA7	-1.2352461	0.0118527			
			POLK	-1.6512044	9.73E-06	CX3CL1	-1.1266509	0.01715521			
			WRN	-1.3719128	1.01E-05	PCDHGB2	-1.1242889	0.02772207			
			SMC5	-1.5896138	1.03E-05	PCDHGA12	-1.0233256	0.03637636			
			FANCM	-1.5768893	1.07E-05						
			BLM	-1.4810655	1.07E-05						

			POLN	-2.0832777	1.13E-05						
			PRIMPOL	-1.4580238	1.16E-05						
			MLH3	-1.2738219	1.71E-05						
			FANCI	-1.0675134	1.84E-05						
			CHEK1	-1.1475694	2.07E-05						
			NEIL3	-1.4865582	2.21E-05						
			SPIDR	-1.2856433	2.22E-05						
			FANCB	-1.6539536	2.80E-05						
			XRCC2	-1.7100603	3.02E-05						
			SMCHD1	-1.0189134	3.98E-05						
			SHPRH	-1.5680241	4.39E-05						
			BOD1L1	-1.3585218	4.43E-05						
			FIGNL1	-1.2604956	5.58E-05						
			LOC100133315	-2.0724603	5.94E-05						
			UIMC1	-1.0809185	7.79E-05						
			CDC45	-1.2659465	8.98E-05						
			EME1	-1.3115028	0.00010809						
			PMS2P3	-1.8204234	0.00011225						
			SETD2	-1.1093902	0.00014652						
			POLG2	-1.2734337	0.00015174						
			DTL	-1.043986	0.00015239						
			POLE	-1.302097	0.00015272						
			RAD54L	-1.3728923	0.00015837						
			CEP164	-1.2816704	0.00015846						
			KIN	-1.1113065	0.00016828						
			XPA	-1.1601856	0.00020277						
			FAN1	-1.0491197	0.00021151						
			SMG1	-1.1105871	0.0002264						
			SMARCAD1	-1.0502345	0.00025649						
			RAD50	-1.0614335	0.00027186						
			UVSSA	-1.1628002	0.00040339						
			MMS22L	-1.1805712	0.00041879						
			INTS3	-1.0449485	0.00067038						
			PARPBP	-1.1479582	0.00106142						
			TICRR	-1.212044	0.00123955						
			DMC1	-1.2899562	0.00255417						
			POLE2	-1.0964404	0.00284171						
			MIR221	-1.2159211	0.01144615						
			DDX11	-1.1152153	0.01424132						
